# Calcium phosphates and silicon: exploring methods of incorporation

**DOI:** 10.1186/s40824-017-0092-8

**Published:** 2017-04-19

**Authors:** Ana I. Rodrigues, Rui L. Reis, Clemens A. van Blitterswijk, Isabel B. Leonor, Pamela Habibović

**Affiliations:** 1 3B’s Research Group – Biomaterials, Biodegradables and Biomimetics, University of Minho, Headquarters of the European Institute of Excellence on Tissue Engineering and Regenerative Medicine, AvePark - Parque de Ciência e Tecnologia, Zona Industrial da Gandra, 4805-017 Barco GMR, Portugal; 2 ICVS/3B’s – PT Government Associate Laboratory, Braga/Guimarães, Portugal; 3 Department of Tissue Regeneration, MIRA Institute, University of Twente, Enschede, The Netherlands; 4 Department of Instructive Biomaterials Engineering, MERLN Institute for Technology-Inspired Regenerative Medicine, Maastricht University, PO Box 616, 6200 MD Maastricht, The Netherlands

**Keywords:** Calcium phosphates, Silicon, Osteogenic differentiation, Human mesenchymal stem cells

## Abstract

**Background:**

Bioinorganics have been explored as additives to ceramic bone graft substitutes with the aim to improve their performance in repair and regeneration of large bone defects. Silicon (Si), an essential trace element involved in the processes related to bone formation and remodeling, was shown not only to enhance osteoblasts proliferation but also to stimulate the differentiation of mesenchymal stem cells (MSCs) and preosteoblasts into the osteogenic lineage. In this study, the added value of Si to calcium phosphate (CaP) coatings was evaluated.

**Methods:**

Tissue culture plastic well plates were coated with a thin CaP layer to which traces amounts of Si were added, either by adsorption or by incorporation through coprecipitation. The physicochemical and structural properties of the coatings were characterized and the dissolution behavior was evaluated. The adsorption/incorporation of Si was successfully achieved and incorporated ions were released from the CaP coatings. Human MSCs were cultured on the coatings to examine the effects of Si on cell proliferation and osteogenic differentiation. For the statistical analysis, a one-way ANOVA with Bonferroni post-hoc test was performed.

**Results:**

The results showed that human MSCs ﻿(hMSCs)﻿ responded to the presence of Si in the CaP coatings, in a dose-dependent manner. An increase in the expression of markers of osteogenic differentiation by human MSCs was observed as a result of the increase in Si concentration.

**Conclusions:**

The incorporation/adsorption of Si into CaP coatings was successfully achieved and hMSCs responded with an increase in osteogenic genes expression with the increase of Si concentration. Furthermore, hMSCs cultured on CaP-I coatings expressed higher levels of ALP and OP, indicating that this may be the preferred method of incorporation of bioinorganics into CaPs.

## Background

Calcium phosphates (CaPs), bioceramics which are found in natural bone mineral, have been extensively used in orthopedic and craniomaxillofacial surgery since the 1970’s owing to their biocompatibility and osteoconductive properties [1, 2]. In the past 15 years, extensive efforts have been invested in tailoring the properties of CaP ceramics, including their mechanical properties and degradation profile [3–5]. Furthermore, in a search for a successful alternative to autografts, being the gold standard in bone regeneration, many researchers have focused on improving bioactivity of CaP ceramics, making them not only osteoconductive but also osteoinductive [6, 7]. To this end biological growth factors with known osteoinductive potential, such as bone morphogenetic proteins (BMPs), have been used as additives to CaP ceramics [8]. While some excellent clinical successes have been achieved with BMP-loaded CaPs [9], the use of BMPs is associated with high costs and stability issues. Therefore, need exists for fully synthetic bone graft substitutes with osteoinductive properties. Recently, CaP ceramics with intrinsic osteoinductivity were developed by optimizing their physico-chemical properties. The use of inorganic ion additives is increasing due to their known role in processes related to bone formation and remodeling [10–12]. Thus, inorganic ions have been used as method to improve bioactivity of CaPs without compromising their synthetic character [13–15].

Several early studies by Carlisle have shown that silicon (Si) has a direct role in bone metabolism and should be considered an essential trace element for metabolic processes occurring in bone [16–18]. Si in aqueous solutions was shown to enhance proliferation of osteoblasts and to increase alkaline phosphatase activity and osteocalcin expression, which are markers of osteogenic differentiation [19, 20]. Also, several in vitro studies with bioactive glass have shown that its dissolution products, rich in Si, stimulated osteogenic differentiation and increased the viability of osteoblasts [21–24]. A number of in vitro studies have explored the biological effects of Si substitution in CaP bioceramics. For example, Hing et al. [25] and Mastrogiacomo et al. [26] have shown that the presence of Si in CaPs, hydroxyapatite (HA) and tricalcium phosphate (TCP) ceramics respectively, increased bone deposition and ingrowth in a femoral defect in New Zealand White rabbits and in long-bone defects in sheep. The presence of Si in alpha-tricalcium phosphate (α-TCP) and HA was also demonstrated to increase osteogenesis in an in vitro model [27] and new matrix formation in a Wistar rat femur model [28] when compared to pure α-TCP or HA. In vivo studies have also shown a more pronounced bone growth inside Si substituted HA [29] as well as a faster bone remodeling around the implant [30]. In a recent study, it was shown that on Si-doped dicalcium phosphate dehydrate (DCPD, brushite) cements a more pronounced resorptive activity of osteoclast-like cells occurred as compared to their undoped counterparts. Furthermore, significantly more bone formation was observed after a 4-week implantation of Si-doped cements in a rat femoral model [31]. In another study, a more stable actin sealing zone was observed in osteoclasts cultured on SiHA as compared to HA, an effect that was suggested to contribute to a more pronounced resorptive activity in the substituted ceramics [32].

In this context, the aim of the current work was to assess the in vitro response of human mesenchymal stem cells (hMSCs) to Si that was added to a CaP coating. To this end, thin CaPs coatings were deposited on tissue culture plastic by employing a biomimetic precipitation method [33]. Si in different concentrations was either adsorbed onto the surface of the coating after precipitation or incorporated into the coating during precipitation. Physicochemical and structural properties of the coatings were fully characterized, as well as the degradation and release profile of the different ions. Finally, hMSCs were cultured on these different coatings, with, as an additional condition, cultures in which Si was directly added to the cell culture medium. Proliferation and osteogenic differentiation of hMSCs at the enzyme and mRNA levels were characterized over a 14-day culture period.

## Methods

### Experimental design

hMSCs were exposed to Si combined with CaP coatings in three different conditions: (1) CaP coating was deposited on tissue culture well plates alone as negative control group, followed by (2) Si adsorption (CaP-A), (3) Si was incorporated into CaP during the crystal growth step of the coating procedure (CaP-I) and (4) Si was added to the culture medium during cell culture on CaP coated well plates (CaP-M). hMSCs were then cultured on the materials during 14 days. Six samples (*n* = 6) were analyzed per condition per time point in two different experiments.

### Preparation of biomimetic calcium phosphate coatings

Biomimetic CaP coatings were deposited in a two-step procedure consisting of precalcification and crystal growth steps as previously described by Yang et al. [33]. Briefly, the precalcification was done by filling the wells of 12-well tissue culture plates (Costar) with a 2.5 times concentrated Simulated Body Fluid (SBF) that was prepared by mixing a “buffer” solution (12.1 g Tris base, 82 ml 1 M HCl in pure MilliQ water to a total volume of 2 L, pH = 7.4), a “calcium” stock solution (25 mM CaCl_2_.2H_2_O, 1.37 M NaCl, 15 mM MgCl_2_.6H_2_O in “buffer” solution) and a “phosphate” stock solution (11.1 mM Na_2_HPO_4_.H_2_O, 42 mM NaHCO_3_ in “buffer” solution) at a ratio of 2:1:1. 500 μl of SBF 2.5x solution was added to each well at 25 °C for 3 days with daily refreshment, resulting in the formation of a thin amorphous CaP coating that acted as nucleation layer for crystal growth of the final coating formed in the second step. For the second step, Calcium Phosphate Solution (CPS) was prepared consisting of 2.25 mM Na_2_HPO_4_.H_2_O, 4 mM CaCl_2_.2H_2_O, 0.14 M NaCl, 50 mM Tris in MilliQ water, pH 7.4). Silicon stock solution (SiS) of 10 mM Si was prepared by dissolving Na_2_SiO_3_ in “buffer” solution as described above.

For the CaP-A group, the CaP-coated well plates were filled with SiS diluted in the “buffer” solution to a final Si concentration of 0, 1, 5 and 10 mM for 4 h at 50 °C. For the CaP-I group, different volumes of SiS were added to CPS to obtain Si concentrations of 0, 1, 5 and 10 mM. CPS containing Si was then added to precalcified plates from the first step at 25 °C for 3 days with daily refreshment, after which the plates were washed with ultrapure water and dried at 50 °C overnight. Before cell culture, all plates were sterilized with 70% ethanol for 20 min, washed with sterile PBS and dried in a sterile hood. Wells were then washed with 500 μL of cell culture medium for 1 h that was discarded prior to cell seeding. For the CaP-M group, the cells were cultured on CaP coated well plates, in a cell culture medium to which Si was added in a concentration of 1 mM. This concentration was selected based on the preliminary data (not shown) indicating that direct addition of the ion in concentrations higher than 5 mM was toxic to cells.

### CaP coating characterization and ion release profile

The CaP coating morphology and the elemental composition were evaluated using a scanning electron microscope (SEM: JSM-6010LV, JEOL, Japan) equipped with energy dispersive spectroscopy (EDS: INCAx-Act, PentaFET Precision, Oxford Instruments, UK). Prior to the SEM evaluation, the plates were dehydrated in a series of ethanol-water solutions with increasing ethanol concentrations (30%, 50%, 70%, 80%, 90%, 95% and 100%, v/v) and dried overnight. Plates were cut and coated with gold–palladium by ion sputtering. A graphite coating was used for the EDS analysis. The SEM and the EDS analyses were performed using three samples for each condition.

The chemical structure of the CaP coatings was analyzed by Fourier-transformed IR spectroscopy (FTIR) in an IRPrestige-21 (Shimadzu, Japan). Analysis was performed using potassium bromide (KBr)-based pellets at a sample:KBr dilution ratio of approximately 1:100. Spectra were collected at 4 cm^**−**1^ resolution using 60 scans in the spectral range 4400– 800 cm^**−**1^. For each sample, three individual measurements were performed.

The concentration of Si in the cell culture medium was determined using inductively coupled plasma optical emission spectroscopy (ICP-OES) by collecting and pooling the samples after 3, 7 and 14 days of culture. Therefore, the results represent a cumulative release profile. The medium solutions were filtered with a 0.22 μm filter and diluted (1:10) in 1% HNO_3_ and kept at -20 °C until use. Basic medium was used as a control.

### In vitro biological response of hMSCs to CaP coatings

#### Cell culture

hMSCs used in this study were isolated from bone marrow aspirates obtained from two healthy donors (females, 66 and 74 year of age, respectively) after written informed consent, according to the protocol described earlier [34, 35]. In short, the cells were thawed, plated at a density of 5.000 cells/cm^2^ and cultured in proliferation medium (PM), consisting of basic medium (BM) (comprising D-MEM (Gibco), 10% fetal bovine serum (Lonza), 2 mM L-glutamine (Gibco), 0.2 mM ascorbic acid (Sigma), 100 U/ml penicillin and 100 mg/ml streptomycin (Gibco)) supplemented with 1 ng/ml recombinant human basic fibroblast growth factor (AbD Serotec). hMSCs were allowed to expand in PM with medium refreshment every 2–3 days.

Cells of the passage 2–3 were seeded on CaP-coated 12-well plates at a density of 10.000 cells/cm^2^ in BM. For the real time quantitative PCR analysis, the cell seeding density was increased to 20.000 cells/cm^2^ in order to obtain sufficient RNA to perform the assay. The cells were incubated for a period of 3, 7 and 14 days in a humidified atmosphere with 5% CO_2_ at 37 °C and the medium was changed every second day. The cells cultured on CaP coatings in the absence of Si in basic or osteogenic medium were used as controls.

#### Cell proliferation assay

In order to assess the proliferation of cells cultured on the different CaP coatings the DNA amounts were quantified using the fluorescent picoGreen double-stranded DNA quantification assay (Invitrogen Corporation, USA). After 3, 7 and 14 days of culture, the samples were collected, rinsed with PBS and frozen at **-**80 °C for at least 24 h. Prior to analysis, the samples were thawed at room temperature and then sonicated for 15 min to induce complete membrane lysis. Supernatant fluorescence was measured (485 nm excitation and 528 nm emission) using a microplate reader (Synergy HT, BioTek Instruments, USA) and the DNA amounts were calculated according to a standard curve. Triplicates were analyzed for each sample at each time point.

#### Quantification of alkaline phosphatase (ALP) activity

ALP activity from the cells cultured on CaP coatings was quantified after 3, 7 and 14 days of culture by the specific conversion of p-nitrophenyl phosphate (pNPP, Sigma) to p-nitrophenol (pNP, Sigma). Prior to analysis, the cells were treated in the same way as described above for the proliferation assay. Then, a buffer solution containing 0.2% (w/v) pNPP was added to the supernatant in a 96-well plate (Costar, Becton Dickinson). The enzyme reaction was carried out at 37 °C for 45 min and then stopped by a solution containing 2 M NaOH and 0.2 mM EDTA in distilled water. The absorbance of pNP formed was read at 405 nm in a microplate reader (Synergy HT, BioTek Instruments, USA). A standard curve was made using pNP values ranging from 0 to 0.2 mmol ml^−1^. ALP activity was normalized to DNA levels.

#### RNA isolation, cDNA synthesis and real time quantitative PCR

Real time qPCR analysis was performed to analyze the relative gene expression of a panel of osteogenic markers upon culture of hMSCs on the coatings for 7 or 14 days. The corresponding osteoblastic genes primer sequences are summarized in Table 1. Total RNA was isolated using the Trizol (Invitrogen) method according to the manufacturer’s protocol. Constructs were washed with PBS, immersed in Trizol and stored at -80 °C until further use. Chloroform was used for protein removal. The RNA pellets were washed with isopropyl alcohol and 70% ethanol and collected in RNAse free water (Gibco, Invitrogen). Quantification was then performed using the Nanodrop ND 1000 spectrophotometer (ThermoScientific) (triplicates of each material per time point were performed).

**Table 1:** Primer sequences

Name	Primer sequences (5’-3’)
ALP	ACAAGCACTCCCACTTCATC TTCAGCTCGTACTGCATGTC
OP	CTCCATTGACTCGAACGACTC CAGGTCTGCGAAACTTCTTAGAT
RUNX2	TGGTTACTGTCATGGCGGGTA TCTCAGATCGTTGAACCTTGCTA
OC	TGAGAGCCCTCACACTCCTC CGCCTGGGTCTCTTCACTAC
BMP2	ACTACCAGAAACGAGTGGGAA GCATCTGTTCTCGGAAAACCT
GAPDH	CGCTCTCTGCTCCTCCTGTT CCATGGTGTCTGAGCGATGT

The cDNA synthesis was performed in the RT-PCR Mastercycler (Realplex, Eppendorf) using the iScript cDNA Synthesis Kit (BioRad) with an initial amount of 1 μg of RNA in a total volume of 20 μL. After the single-strand cDNA synthesis, the target cDNA was amplified for real-time PCR quantification according to the manufacturer’s protocol. Cycles of denaturation, annealing and extension were carried out in the gradient thermocycler MiniOpticon real-time PCR detection system (BioRad) for all genes. The expression of the osteogenic marker genes was normalized to the housekeeping gene glyceraldehyde-3-phosphate-dehydrogenase (GAPDH) levels and fold induction were calculated using the comparative ΔCT method.

### Statistical analysis

All the quantitative results were obtained from triplicate samples. Data are reported as mean ± standard deviation. For the statistical analysis, a one-way ANOVA with Bonferroni post-hoc test was performed, and the differences were considered statistically significant when *p* < 0.05.

## Results

### CaP coating characterization and ion release profile

**Fig. 1:**
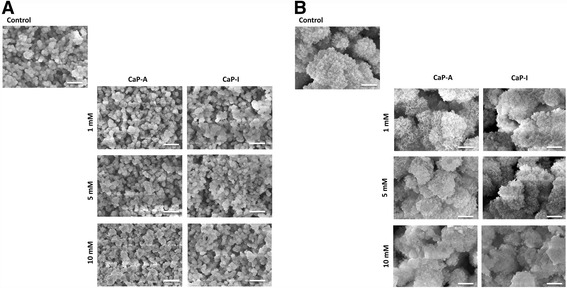
Scanning electron micrographs of the calcium phosphate coatings at (**a**) × 2000 magnification (scale bar corresponds to 10 μm) and (**b**) × 10000 magnification (scale bar corresponds to 1 μm). Low magnification images showed a homogeneous deposition of the mineral coating in all conditions. Higher magnification images revealed less sharp crystals with the incorporation of higher Si concentrations

**Fig. 2:**
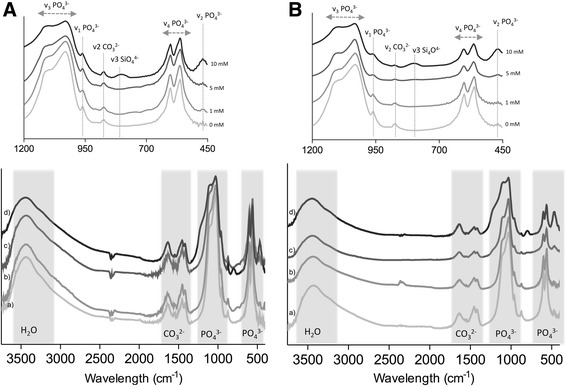
FTIR spectrum of CaP-A (**a**) and CaP-I (**b**) coatings made with (a) 0, (b) 1, (c) 5 and (d) 10 mM of Si. The main PO_4_^3−^ and CO_3_^2−^ groups and peaks are indicated. Results showed a decrease in H_2_O and PO_4_^3−^ groups with the increase in Si content. Furthermore, the presence of SiO_4_^4−^ was also observed in both CaP-A and CaP-I with 10 mM of Si

The morphology and the surface elemental analysis of the CaP coatings used in this work were evaluated by SEM and EDS, respectively. The low magnification SEM images (Fig. 1a) showed that a homogenous mineral coating was deposited on the surface of tissue culture well plates in all conditions. There was no obvious effect of the presence of Si in the CPS solution on the homogeneity of the coating. Similarly, the adsorption process of Si after the precipitation of CaP coating did not affect its homogeneity. All coatings consisted of CaP globules with a size of 2-4 μm, without obvious differences among different conditions. Higher magnification SEM images (Fig. 1b), however, revealed differences in the surface morphology of the coating globules. While in the control coating, and in the coating containing low concentration of incorporated Si, sharp crystals, oriented perpendicular to the coating surface were observed, incorporation of higher Si concentrations affected the coating morphology, making the crystals less sharp. Also, the adsorption process of Si on the surface affected the surface by decreasing the size and the sharpness of the crystals. FTIR results for both adsorbed (Fig. 2a) and incorporated (Fig. 2b) Si showed a decrease in the band intensities corresponding to O-H and P-O (most noticeable around 962 cm^−2^) as the Si content increased. Also, in the coatings spectra of 10 mM Si adsorbed and incorporated conditions, a band around 810 cm^−1^ corresponding to v3 Si_4_O^4−^ is visible. In both coating processes, a spectrum that is typical of HA like phase with main PO_4_^3−^ peak in v1 mode around 960 cm^−2^ was observed. Shifting of v1 PO_4_^3−^ peak to lower frequency indicated a reduced crystallinity in comparison to the control.

EDS analysis (Table 2) showed that the increase in the Si concentration, either through incorporation during the coating process or through adsorption on the surface, from 0 to 10 mM, resulted in the increase of atomic percentage of Si in the respective coatings. Furthermore, the atomic percentage of Si at higher concentrations was higher in the CaP-I than in CaP-A group.

**Table 2:** EDS analysis of CaP coatingsData are shown as mean ± standard deviation from *n* = 3 samples

		O (at%)	Ca (at%)	P (at%)	Si (at%)
Control		45,33 ± 0,60	37,77 ± 0,46	16,87 ± 0,25	
CaP-A	1 mM	45,6 ± 0,35	36,1 ± 0,14	16,8 ± 0,42	1,50 ± 0,35
	5 mM	42,95 ± 0,35	39,05 ± 0,07	16,00 ± 0,07	2,00 ± 0,07
	10 mM	44,6 ± 0,61	34,67 ± 0,85	12,73 ± 0,64	7,97 ± 0,89
	1 mM	46,75 ± 0,07	35,95 ± 0,07	16,25 ± 0,07	1,00 ± 0,14
CaP-I	5 mM	42,83 ± 2,01	31,6 ± 2,18	14,07 ± 0,55	11,53 ± 0,59
	10 mM	51,35 ± 0,35	21,6 ± 1,27	9,05 ± 0,64	17,9 ± 1,56

**Fig. 3:**
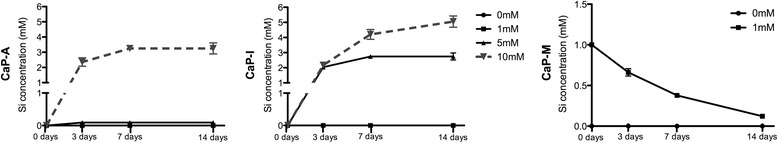
Elemental concentration of silicon (Si) in cell culture medium after 3, 7 and 14 days of incubation in presence of CaP-A and CaP-I coatings made with 0, 1, 5 and 10 mM of Si and CaP-M coatings with 0 and 1 mM of Si. Data are shown as mean ± standard deviation from *n* = 3 samples. Results showed an increase in Si concentration in the cultured medium of CaP-A 10 mM and CaP-I 5 and mM. Cell culture medium from CaP-M revealed a decrease in Si concentration after 14 days

The release profile of Si from the CaP coatings into the culture medium during cell culture was evaluated using the ICP-OES (Fig. 3). In the CaP-A group, an increase in Si concentration in the culture medium was observed for CaP-A 10 mM coatings, from 0 mM to approximately 3.1 mM during 7 days of culture after which the concentration remained constant until day 14. The values found for other CaP-A conditions were close to 0 mM at all time points. In the CaP-I group, the coating prepared with 5 mM in the solution, showed an increase in Si concentration in the cell culture medium up to a level of approximately 2.1 mM at 7 days, after which the concentration remained constant. In the CaP-I Si 10 mM, a continuous increase in concentration in time was observed up to a concentration of 4.8 mM at day 14. The values found for CaP-I 1 mM were close to 0 mM at all time points. In the CaP-M group, where Si was added to cell culture medium, a decrease in Si concentration in time was observed from 1 mM to approximately 0.1 mM after 14 days.

### hMSCs proliferation

**Fig. 4:**
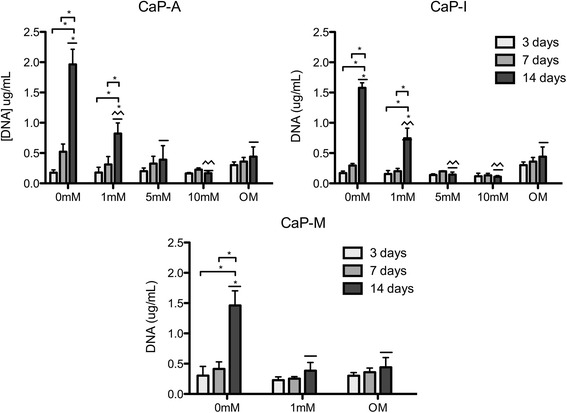
DNA quantification of hMSC cultured on CaP-A, CaP-I with 0, 1, 5 and 10 mM of Si and CaP-M with 0, and 1 mM of Si after 3, 7 and 14 days. OM represents the condition in which the cells were cultured on CaP coatings in osteogenic medium. Significant differences among different conditions analyzed at the same time point are indicated by asterisks + patterned lines. Connector lines + asterisk indicate the significant differences in the same condition between different time points. (*p* < 0.05; *n* = 6). CaP coatings without and on CaP-A and CaP-I with 1 mM sustained cell proliferation. For the other conditions, DNA values did not present significant variances

Cell proliferation was evaluated by quantifying total DNA amounts after 3, 7 and 14 days of culture of hMSCs on different coatings (Fig. 4). An increase in DNA amounts in time was observed on CaP coatings without, and on CaP-A and CaP-I coatings with 1 mM Si. In all other conditions, including cell culture on CaP coating without Si in OM, DNA amounts remained constant or only slightly changed in time.

### ALP activity

**Fig. 5:**
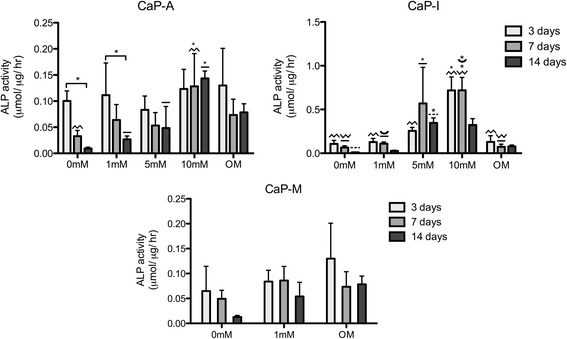
ALP activity of hMSC cultured on CaP-A, CaP-I with 0, 1, 5 and 10 mM of Si and CaP-M with 0, and 1 mM of Si after 3, 7 and 14 days. OM represent the condition in which the cells were cultured on CaP coatings in osteogenic medium. Significant differences among different conditions analyzed at the same time point are indicated by asterisks + patterned lines. Connector lines + asterisk indicate the significant differences in the same condition between different time points. (*p* < 0.05; *n* = 6). Values were normalized to μg of DNA. ALP expression was remarkably higher in CaP-I coatings. Cells cultured on CaP-I 5 mM and 10 mM showed a higher ALP activity than the other conditions. CaP-A 10 mM showed a higher ALP expression than CaP-A 1 mM and 5 mM Si after 14 days of culture

The ALP activity was quantified after 3, 7 and 14 days of culture (Fig. 5). Cells cultured on CaP-I coatings showed a higher ALP activity as compared to the cells cultured on either CaP-A or CaP-M coatings. Cells cultured on CaP-A 10 mM showed a significantly higher ALP expression as compared to the coating without Si after 7 days of culture, and than the coatings prepared with 1 mM and 5 mM Si after 14 days. In the CaP-I group, a higher activity of ALP was observed in presence of higher Si concentration, as compared to the coating prepared without and with 1 mM Si. Cells cultured on CaP-A 0 mM and 1 mM showed a significant decrease in ALP activity in time. In other conditions, no significant temporal changes in ALP activity were observed.

### Gene expression

**Fig. 6:**
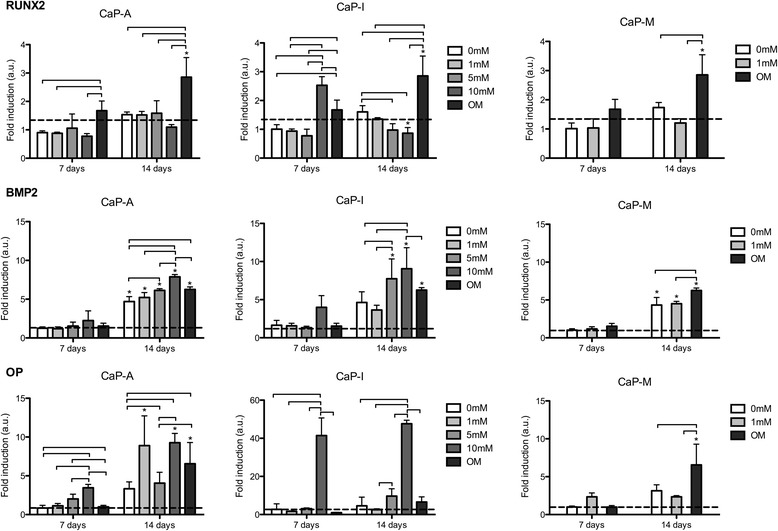
Expression of osteogenic markers at mRNA level of hMSC seeded on CaP-A, CaP-I with 0, 1, 5 and 10 mM of Si and CaP-M with 0, and 1 mM of Si after 3, 7 and 14 days. OM represents the condition in which the cells were cultured on CaP coatings in osteogenic medium. Significant differences among the same condition analyzed at different time points are indicated by asterisks. Connector lines indicate the significant differences between different conditions at the same time point. (*p* < 0.05; *n* = 6). RUNX2 expression was significantly higher in CaP-I 10 mM than in the other conditions at 7 days of culture. BMP2 was up-regulated in CaP-A and CaP-I 5 and 10 mM as well as for CaP-M in osteogenic medium. Similar behavior was found for OC. OP was significantly up-regulated in both CaP-A and CaP-I 10 mM, a slight increase was also observed for Ca-A and CaP-I 5 mM. All conditions presented an up-regulation of the studied genes from day 7 to day 14

QPCR data showing the expression of a panel of osteogenic markers at mRNA level of hMSCs cultured on different coatings are presented in Fig. 6. In the CaP-A group, the expression of RUNX2 was not affected by the presence of Si at either time point. The only effect observed was an upregulation of the RUNX2 expression when cells were cultured on CaP coatings without Si in osteogenic cell culture medium. A similar effect was observed in the CaP-I and CaP-M group, with the exception of CaP-I 10 mM at day 7 that exhibited a higher RUNX2 expression as compared to the other conditions.

Regarding the mRNA expression of BMP2, in both CaP-A and CaP-I groups, both 5 mM Si and 10 mM Si condition showed significantly higher values than CaP-A and CaP-I 0 and 1 mM. In both groups, the 10 mM condition showed a higher BMP2 expression as compared to that of cells cultured in osteogenic medium. Cells cultured in osteogenic medium had a significantly higher expression than the 0 and 1 mM condition of the CaP-M group. In all conditions, an up-regulation in BMP2 expression from day 7 to day 14 was observed.

A trend similar to that of the BMP2 expression was observed for the expression of OC. At 14 days, cells cultured in 5 and 10 mM Si condition of both CaP-A and CaP-I groups had a significantly higher OC expression than all other conditions in the respective group. At 7 days, significant differences were observed between CaP-A 10 mM and both 0 mM and OM and between CaP-M 1 mM when compared to 0 mM. Again, all the conditions showed an upregulation in OC expression from day 7 to day 14.

The expression of OP was significantly upregulated in the presence of 10 mM Si, in both CaP-A and CaP-I groups, as compared to the other conditions at both time points. A mild positive effect was also seen for the 5 mM condition. In the CaP-M groups, the presence Si did not have a positive effect, and only the type of medium affected the expression of OP after 14 days of culture. A significant temporal increase in OP expression was observed in CaP-A 1 mM, 10 mM and OM and CaP-M OM.

## Discussion

The interaction between a degradable bone graft substitute and the tissue at the implant site is governed by a continuous process of ions migration and consequent changes in ionic concentration. It is hypothesized that bone induction by Si-containing materials can occur through direct contact between the cells and the material surface as well as through the release of soluble ions during degradation processes. During the process of deposition of the mineralized bone layer on the surface of the implant, the co-precipitation of salts and ions is expected to occur. To better understand this process, Patntirapong et al. [11] have examined the in vitro response of osteoclasts to cobalt (Co) ions, delivered to cell culture medium or incorporated/adsorbed into a CaP coating. They demonstrated that the incorporation/adsorption of Co was successful and did not affect the growth mechanism of CaP crystal growth, allowing to study the effects of Co ions on the growth and resorptive activity of osteoclasts. Following the same rationale, in the current study, CaP coatings were combined with different concentrations of Si using the methods of adsorption, incorporation or direct addition to the culture medium, and the effect of Si addition on proliferation and osteogenic differentiation of hMSCs was studied in vitro. The approach consisted of coating tissue culture multi-well plates with thin CaP layers containing different concentration of Si, using three different methods.

Previous studies [11, 33] have shown that by using the biomimetic method of precipitation of CaP inside tissue culture well plates, a homogenous layer, consisting of a mix of an octacalcium phosphate and an apatitic phase is formed. In the present study, the formation of a homogenous mineral layer was also observed as was shown by the low-magnification SEM images, without obvious effect of Si concentration. However, higher-magnification SEM images exhibited a reduction in crystal size and sharpness in the presence of higher concentrations of Si, when compared to the control. This was in accordance with a previous report [36], showing that an increase in Si concentration incorporated by an aqueous precipitation method resulted in a decrease in crystal size.

The incorporation/adsorption of Si in the coatings was demonstrated by the FTIR spectra showing a decrease in O-H and P-O bands intensities with the increase of Si concentration and the presence of the v_3_ Si_4_O^4−^ band in 10 mM concentration of both adsorbed and incorporated Si. These results are in accordance with semi-quantitative EDS data showing an increase in Si atomic percentage with the increase in adsorbed or incorporated Si concentration.

It was previously stated that a close correlation exists between the amount of silicon incorporated into HA ceramics and their dissolution rate [36]. Therefore, we studied the release profile of Si from the different materials. Regarding the evaluation of the release profile of Si in the cell culture medium, CaP-A 10 mM, CaP-I 5 mM and 10 mM enriched medium with Si in a continuous manner during 7 days of culture, after which the concentrations remained constant. In the cell culture medium of CaP-M samples, as expected, a decrease in Si concentration during the culture time was observed, indicating that precipitation of Si onto the CaP layer occurred. This was in accordance with a previous study [11] where a decrease of Co^2+^ concentration in the medium was observed, 3 days after cell seeding. The Si release studies denoted that the concentration of Si in the medium, for all the conditions, was below 6 mM, a value that has been shown to be toxic to cells [37]. This demonstrates that, despite the fact that solutions with higher Si concentrations were used for the incorporation/adsorption, the released amount of Si was considerably lower, showing that the control of Si release from the CaP is highly important and needs a deeper understanding.

Cell proliferation results demonstrated a clear difference between the different concentrations of Si used in our study, however no relevant differences were observed among the three addition methods used. Cells cultured with 0 mM and 1 mM Si significantly proliferated over a period of 14 days and showed high DNA amounts. In contrast, DNA amounts of cells cultured with 5 mM and 10 mM Si, as well as those cultured in osteogenic medium, remained constant and were lower than the conditions without or with low Si concentrations. While cell proliferation was negatively affected by the presence of higher concentrations of Si, ALP activity of cells cultured in presence of materials containing higher Si concentrations was enhanced as compared with the materials without or with low concentrations of Si. Low proliferation rate of cells cultured at higher Si concentrations may be related to the fact they were undergoing the process of differentiation, as was shown previously [38]. It should be noted that ALP activity of cells cultured in the CaP-I condition was significantly higher than in CaP-A and CaP-M conditions, which may be a consequence of the higher Si release in the former condition, as was demonstrated by the ICP-OES results. It is also important to note that ALP activity values measured in CaP-I and CaP-A 5 mM and 10 mM conditions were close to those measured for the positive control, i.e. cells cultured on the coatings in osteogenic medium. According to several authors [10, 39, 40], calcium and inorganic phosphate ions have a positive effect on the osteogenic differentiation of hMSCs. For example, Danoux et al. [10] demonstrated that culture medium supplemented with 4 or 8 mM Ca^2+^, or 4 mM Pi ions resulted in the enhancement of ALP activity of hMSCs. Therefore, the presence of significant amounts of Ca and P in our coatings, as shown by EDS, and their influence on osteogenic differentiation and, consequently, ALP production may cover the possible additive effects of Si when present in lower concentration (1 mM).

Gene expression analysis revealed that hMSCs expressed significantly higher levels of OC and OP when cultured on CaP coatings with 10 mM Si as compared to other conditions. These are valuable markers of osteogenic differentiation, which play an important role in the regulation of formation and growth of HA crystals during bone mineralization [41, 42]. Also, the presence of 5 mM and 10 mM Si, induced an up-regulation of BMP2, which plays a role in the maintenance of bone homeostasis and regeneration [43]. A study by Honda et al. [44] also demonstrated an increase in OC by osteoblasts cultured on Si substituted HA when compared to HA only. In the same study, an increase in the RUNX2 expression was also observed, which was in contrast to the present study, which may be explained by the difference in cell type used for the study.

The results further showed that adsorption and incorporation of Si at a concentration of 5 mM and 10 mM had a stronger effect on the expression of osteogenic markers than the lower concentrations indicating that Si had a dose-dependent effect. This was in accordance with previous studies, also showing a dose-dependent effect of Si, released from Si-containing materials, on osteoblasts proliferation, osteogenic expression and bone remodeling [19–22, 45].

The incorporation/adsorption of Si into CaP coatings reveals to be a promising strategy to induce osteogenic differentiation of hMSCs. By incorporating/adsorbing higher concentrations of Si we were able to stimulate hMSCs differentiation into the osteogenic lineage without strongly compromising cell proliferation. Our results demonstrated that the incorporation/adsorption of higher concentrations of Si may provide an added value to CaP coatings, which can be applied on geometrically complex shapes and which are suitable for various CaP phases, including thermally less stable ones.

## Conclusions

The incorporation/adsorption of Si into CaP coatings was successfully achieved and the Si ions were released into the cell culture medium. hMSCs responded to the presence of Si at different concentrations in distinct ways, with an increase in osteogenic genes expression with the increase of Si concentration. Furthermore, hMSCs cultured on CaP-I coatings expressed higher levels of ALP and OP, indicating that this may be the preferred method of incorporation of bioinorganics into CaPs.

## Abbreviations

ALP: Alkaline phosphatase
BM: Basic medium
BMPs: Bone morphogenetic proteins
CaP: Calcium phosphate
CaP-A: Calcium phosphate with Si adsorption
CaP-I: Calcium phosphate with Si incorporation
CaP-M: Calcium phosphate with Si added to the medium
CPS: Calcium phosphate solution
DCPD: Dicalcium phosphate dehydrate
EDS: Energy dispersive spectroscopy
FTIR: Fourier-transformed infrared spectroscopy
GAPDH: Glyceraldehyde-3-phosphate-dehydrogenase
HA: Hydroxyapatite
hMSCs: Human mesenchymal stem cells
ICP-OES: Inductively coupled plasma optical emission spectroscopy
MSCs: Mesenchymal stem cells
PM: Proliferation medium
pNP: p-nitrophenol
pNPP: p-nitrophenyl phosphate
SBF: Simulated body fluid
SEM: Scanning electron microscope
Si: Silicon
SiS: Silicon stock solution
TCP: Tricalcium phosphate
α-TCP: Alpha-tricalcium phosphate
